# Selective Detection of Formaldehyde Gas Using a Cd-Doped TiO_2_-SnO_2_ Sensor

**DOI:** 10.3390/s91109029

**Published:** 2009-11-13

**Authors:** Wen Zeng, Tianmo Liu, Zhongchang Wang, Susumu Tsukimoto, Mitsuhiro Saito, Yuichi Ikuhara

**Affiliations:** 1 College of Materials Science and Engineering, Chongqing University, Chongqing 400044, P. R. China; E-Mail: zeng_wen1982@yahoo.com.cn; 2 World Premier International Research Center, Advanced Institute for Materials Research, Tohoku University, 2-1-1 Katahira, Aoba-ku, Sendai 980-8577, Japan; E-Mails: tsukimoto@wpi-aimr.tohoku.ac.jp (S.T.); saito@wpi-aimr.tohoku.ac.jp (M.S.); ikuhara@sigma.t.u-tokyo.ac.jp (Y.I.)

**Keywords:** volatile organic compound, formaldehyde, TiO_2_-SnO_2_, Cd doping, gas sensor

## Abstract

We report the microstructure and gas-sensing properties of a nonequilibrium TiO_2_-SnO_2_ solid solution prepared by the sol-gel method. In particular, we focus on the effect of Cd doping on the sensing behavior of the TiO_2_-SnO_2_ sensor. Of all volatile organic compound gases examined, the sensor with Cd doping exhibits exclusive selectivity as well as high sensitivity to formaldehyde, a main harmful indoor gas. The key gas-sensing quantities, maximum sensitivity, optimal working temperature, and response and recovery time, are found to meet the basic industrial needs. This makes the Cd-doped TiO_2_-SnO_2_ composite a promising sensor material for detecting the formaldehyde gas.

## 1. Introduction

The evaluation of indoor air quality is serious environmental issue that is urgently in need of being addressed. Indoor pollutants, which mainly consist of volatile organic compounds (VOCs) such as formaldehyde, benzene, toluene, xylene, and methanol, are well known to enable the so-called building-related sickness [1]. The conventional method used for monitoring indoor VOCs is generally time-consuming and expensive because it involves on-site sampling of indoor air but ensuing analysis of the sampled air in a laboratory [2–5]. To date, there are still no electronic sensors available for *in situ* detection of indoor VOCs, in particular, the formaldehyde [6], which has already been classified as a probable carcinogen [7–9]. For this reason, extensive research has been conducted on developing a sensor that has a simple device structure and can be put into practical use. To this end, a number of potential sensing materials have been fabricated through a variety of methods [10–12].

Of all the materials investigated, semiconductor metal oxides are promising for monitoring the harmful VOCs owing to their various advantages, for example, simple fabrication process, rapid response and recovery, and low cost. In particular, the oxides SnO_2_ and ZnO, are of strong current interest because they enable an effective detection of VOC gases, especially formaldehyde [13–15]. However, one of the critical issues currently limiting the wide use of these oxides is their lack of selectivity towards formaldehyde [16]. Therefore, in this work, we propose a new nonequilibrium solid solution, TiO_2_-SnO_2_ composite, in order to improve selectivity as well as sensor response to formaldehyde gas. In particular, we introduce Cd into the TiO_2_-SnO_2_ composite and investigate how the Cd doping affects gas-sensing properties. This composite material has been selected deliberately because it is suggested that composite materials may exhibit unusual sensing functionality that is absent in either of their host components [17]. As expected, we find that the Cd-doped TiO_2_-SnO_2_ shows an exclusive selectivity to formaldehyde gas, thereby holding technological promise for fabrication of formaldehyde gas sensors.

## 2. Experimental Procedure

**Figure 1.**
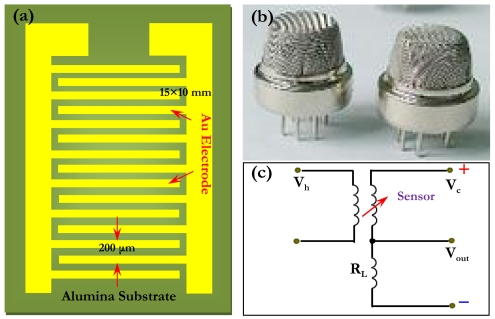
Schematic of a thick-film gas sensor.

The TiO_2_-SnO_2_ solid powder was prepared using the sol-gel method. First, metal salt precursors were hydrolyzed in a dilute pH 3∼5 solution of ammonium hydroxide. Next, the generated metal salt SnCl_4_·5H_2_O and tetrabutyl Ti were dissolved in distilled water. The concentration of Ti cation varied from 0.1 mol/L to 0.2 mol/L, but the ratio of Ti to Sn was maintained at 1:5. To examine the Cd-doping effect, the compound CdNO_2_ was added to the mixed solution in a drop-by-drop fashion under intense magnetic stirring. The mass ratio of Cd additive to total metallic ions was estimated to be about 1 ∼ 2%. It should be noted that as an initial step, we did not address the potential effect of the mass ratio of Cd dopant on gas-sensing properties. To basify the mixed solution, ammonium hydroxide was gradually added dropwise until its ultimate pH reaches 8, which results in immediate precipitation. After the precipitation, the slurry was first aged for 24 h, filtrated and washed to remove chloride ions, then dried at 353 K and ground to a uniform powder. The powder was finally annealed at 723 K for 2 h. For the purpose of comparison, we also prepared pristine TiO_2_-SnO_2_ powder using a similar preparation approach. The produced powder was further mixed with diethanolamine and water to form a paste, which was subsequently screen-printed onto an alumina substrate. The substrate is schematically illustrated in Figure 1(a). A set of comb-like Au electrodes were attached at a distance of 200 μm. The cross-section dimension of the electrodes was fixed at 15 mm × 10 mm and the potential effect of electrode dimension on sensor response was not addressed in present study. The printed substrate was finally sintered at 773 K for 3 h, yielding a thick-film gas sensor [Figure 1(b)]. The film thickness after the sintering was estimated to be about 10∼20 μm.

The microstructures of the solid powder and thick film were characterized using X-ray diffraction (XRD) and atomic force microscopy (AFM), respectively. For the XRD, an XD-5A diffractometry with Cu *K*α operated at 30 kV and 100 mA was used. As for the AFM, we applied a high-speed CSPM4000 microscopy with contact mode, which accurately images surface topography. Gas-sensing properties were measured using a static system controlled by a computer. We used a micro-injector to introduce the VOCs into the chamber and manipulate the VOC concentrations via tuning the input VOC amount alone due to the fixed chamber volume. During the measurement, the sensor was powered at 373 K for 120 h in air and operated at 303 K under a relative humidity of 40%. The gas sensitivity was defined as a ratio of resistance (R_0_) in air to that in test gas (R). As for the measurement of voltage, we adopted a circuit shown in Figure 1(c), which could be divided into a heating and measuring part. Clearly, the output voltage varied with the type and concentration of test gas.

## 3. Results and Discussion

### 3.1. Composition and Microstructure of Gas Sensing Materials

**Figure 2.**
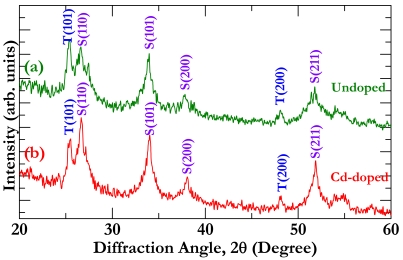
XRD spectra for (a) undoped and (b) Cd-doped TiO_2_-SnO_2_ powder. Note that the T represents TiO_2_ and the S represents SnO_2_.

To determine chemical composition of the prepared powder, we performed XRD analysis, as shown in Figure 2, where textural orientations of the detected matters are given as well for easy reference. From Figure 2(a), one can clearly see TiO_2_ and SnO_2_ peaks in the undoped case, as expected from the aforementioned preparation process. However, no Cd related diffraction peak is detected in the doped case [Figure 2(b)], which is mainly attributed to the small amount of Cd we doped. In light of the approximate relationship between mean particle size (*D*) and full width at high maximum of XRD peak *β* (*i.e.*, Scherrer equation) [18]: *D* = 0.89λ/(*β*cosθ), where λ is the X-ray wavelength (1.541 Å for Cu) and θ is the Bragg angle, the mean particle sizes for the undoped and doped samples were estimated to be about 32 nm and 30 nm, respectively. This means that the Cd doping has a negligible effect on the particle size of the TiO_2_-SnO_2_ composite.

**Figure 3.**
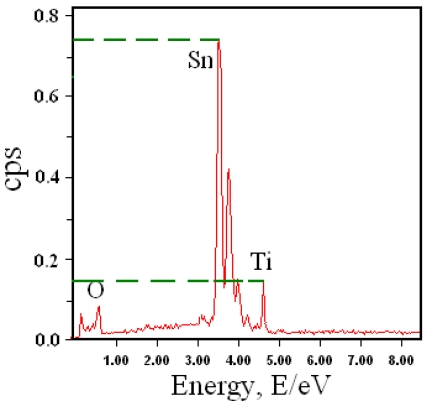
EDS spectrum of the TiO_2_-SnO_2_ thick film.

**Figure 4.**
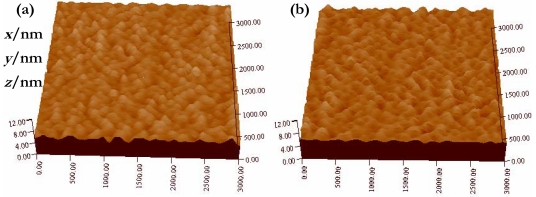
AFM images for (a) pristine and (b) Cd-doped thick films.

To analyze the elemental species of the thick film, we further show in Figure 3 the energy-dispersive X-ray spectroscopy (EDS) spectrum. We notice that the film is mainly composed of Ti, Sn, and O, in accordance with chemical composition of the prepared powder. The mass ratio of Ti to Sn is estimated to be about 1:5 (Figure 3), demonstrating that we have successfully synthesized the desired composite. Figure 4 shows an AFM image of the surface morphology for both the pristine and doped thick film. Many uniform islands can be observed on the surface, which shows that both films have good crystal shape and even grain size. In comparing the surface morphology of pristine TiO_2_-SnO_2_ film with that of doped one, we see that they are similar, which means that the Cd doping affects surface morphology only slightly.

### 3.2. Gas Sensing Properties

**Figure 5.**
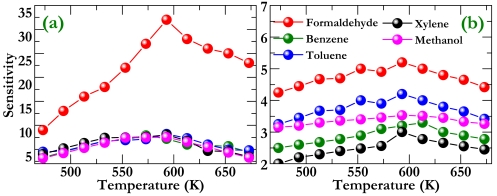
Sensitivity of the sensor fabricated using (a) Cd-doped and (b) pristine TiO_2_-SnO_2_ powder under a gas concentration of 200 ppm. Note that the sensitivity scale for the doped case is much larger than that for the undoped case.

To investigate further how the doping influences sensing properties, we tested the responses of the pristine and doped sensors to different types of VOC gases such as formaldehyde, benzene, toluene, xylene, and methanol. Figure 5(a) shows gas sensitivities under various temperatures, where one can see that the doped sensor exhibits exclusive selectivity to formaldehyde. It should be noted that an understanding of the underlying origin of this exclusive selectivity has not been developed yet, which will be an important future task. The highest sensitivity to the formaldehyde is estimated to be 32, much higher than that to other examined gases (less than 10). Evidently, this demonstrates that the Cd-doped sensor shows good selectivity to formaldehyde, which is therefore promising for practical device applications. From this figure, we further determine the optimum operating temperature to be about 593 K because maximum sensor response to the formaldehyde is observed at this temperature. This can be understood by considering that a dynamic equilibrium state will occur between the initial adsorption and the subsequent desorption, as the operating temperature keeps increasing. The equilibrium state therefore gives rise to a maximum sensitivity, as seen in Figure 5. Compared to the doped case [Figure 5(a)], the sensitivity of pristine sensor is much lower with a maximum value of less than 5 [Figure 5(b)], although the pristine sensor retains the highest sensor response to formaldehyde. We thus conclude that the Cd is an effective dopant in improving response and selectivity of TiO_2_-SnO_2_ composite to formaldehyde.

**Figure 6.**
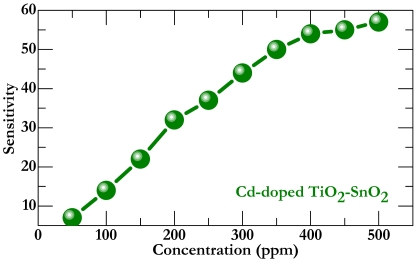
Variation of sensitivity as a function of formaldehyde gas concentration for the Cd-doped sensor. The operating temperature is maintained at 593 K.

Apart from testing the selectivity, we have examined sensitivity of doped sensor as a function of gas concentration. Figure 6 shows sensitivity to the formaldehyde under various concentrations at 593 K. Clearly, the sensitivity increases sharply as the gas concentration ranges from 50 ppm to 450 ppm but saturates when the concentration increases further. It is worth noting that a sensitivity corresponding to concentration of 100 ppm has already reached a value of more than 15, a criterion required for practical application.

**Figure 7.**
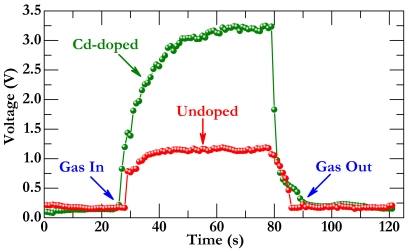
Response-recovery property for sensors fabricated using undoped and Cd-doped composite materials.

Figure 7 presents a representative response-recovery characteristic for the sensor operated at 593 K under a formaldehyde gas concentration of 200 ppm. The response and recovery times are two key quantities for a sensor, which are defined as the time needed to reach 90% response (recovery) when gas is in (out). As seen in this figure, voltage for both the undoped and doped sensor increases sharply when gas is in but returns to its original state while gas is out. The major difference between the two cases is that the voltage for the doped sensor is substantially larger than that for the pristine one at working stage, verifying again significant effect of Cd doping on sensor response improvement. In light of the definition describe above, the response and recovery times for the doped sensor are evaluated to be about 25 s and 17 s, respectively, which meet the basic demands for an industrial application. These short times, together with the high sensor response and exclusive selectivity, suggest that the Cd-doped TiO_2_-SnO_2_ may hold the potential for developing a formaldehyde gas sensor.

### 3.3. Gas Sensing Mechanism

Although many works have been conducted on TiO_2_ or SnO_2_-based sensor, its gas-sensing mechanism remains controversial. The current understanding of the sensing behaviors of TiO_2_ (SnO_2_) material, which is based mainly on experimental studies via a trial-and-error design fashion, can be summarized in three main points: (1) gas sensing process is dominantly controlled by the surface of materials and how the surface chemically absorbs oxygen, (2) once target gases, for example VOC gases, are introduced, oxidization reaction takes place on sensor surface in the following way:

$$\text{VOC}+{\text{O}}^{-}\to \text{VOC}-\text{O}+{\text{e}}^{-};\text{VOC}+{\text{O}}^{2-}\to \text{VOC}-\text{O}+2{\text{e}}^{-}$$

**Figure 8.**
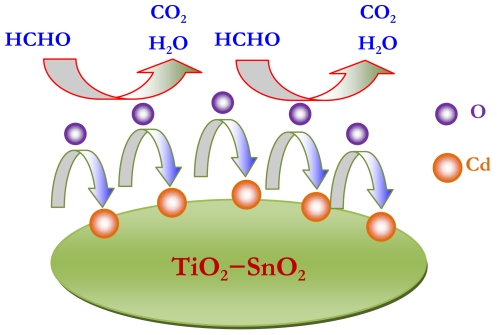
Schematic plot illustrating the sensing mechanism of the Cd-doped sensor. Note that the Cd and O represent the interaction between Cd additives and pre-absorbed oxygen atoms.

(3) gas sensor response may depend critically on the amount of pre-absorbed oxygen. The results presented in this paper demonstrate that the Cd-doped TiO_2_-SnO_2_ material has a significantly higher sensor response to formaldehyde than the pristine material. This enhancement of sensor response can be presumably attributed to the Cd additive, which may provide additional sites for adsorbing oxygen. Consequently, as reductive gas is introduced (Figure 8), more oxidization occurs probably on the Cd surface, which generates more electrons onto the TiO_2_-SnO_2_ surface. These electrons would enhance surface conductivity noticeably, which can therefore reduce surface resistance considerably [19–22], as observed in the response and recovery curves (Figure 7).

## 4. Conclusions

We have applied a sol-gel method to fabricate nonequilibrium TiO_2_-SnO_2_ solid solutions and investigated their microstructures and gas-sensing properties. In particular, we have focused on the effect of Cd doping on gas-sensing properties. We have found that doping is essential for improving both the sensitivity and selectivity of the TiO_2_-SnO_2_-based sensor towards formaldehyde gas. The maximum sensitivity for the doped sensor is found to be 32 under a formaldehyde gas concentration of 200 ppm and the optimum operating temperature to be 593 K. Moreover, the response and recovery times are estimated to be 25 s and 17 s, respectively. These findings demonstrate the potential use of the Cd-doped TiO_2_-SnO_2_ as a formaldehyde gas-sensing material, and this paper presents a possibility for increasing the selection of materials available for other types of gas sensors.
